# New insights into the phosphorylation of the threonine motif of the β1 integrin cytoplasmic domain

**DOI:** 10.26508/lsa.202101301

**Published:** 2022-01-07

**Authors:** Ralph T Böttcher, Nico Strohmeyer, Jonas Aretz, Reinhard Fässler

**Affiliations:** 1 Department of Molecular Medicine, Max Planck Institute of Biochemistry, Martinsried, Germany; 2 Department of Biosystems Science and Engineering, Eidgenössische Technische Hochschule Zurich, Basel, Switzerland

## Abstract

Integrins require an activation step before ligand binding and signaling that is mediated by talin and kindlin binding to the β integrin cytosolic domain (β-tail). Conflicting reports exist about the contribution of phosphorylation of a conserved threonine motif in the β1-tail (β1-pT788/pT789) to integrin activation. We show that widely used and commercially available antibodies against β1-pT788/pT789 integrin do not detect specific β1-pT788/pT789 integrin signals in immunoblots of several human and mouse cell lysates but bind bi-phosphorylated threonine residues in numerous proteins, which were identified by mass spectrometry experiments. Furthermore, we found that fibroblasts and epithelial cells expressing the phospho-mimicking β1-TT788/789DD integrin failed to activate β1 integrins and displayed reduced integrin ligand binding, adhesion initiation and cell spreading. These cellular defects are specifically caused by the inability of kindlin to bind β1-tail polypeptides carrying a phosphorylated threonine motif or phospho-mimicking TT788/789DD substitutions. Our findings indicate that the double-threonine motif in β1-class integrins is not a major phosphorylation site but if phosphorylated would curb integrin function.

## Abstract (precis)

This study investigates phosphorylation of and kindlin recruitment by the double-threonine motif in the β1 integrin cytoplasmic tails.

## Introduction

**Figure 1.**
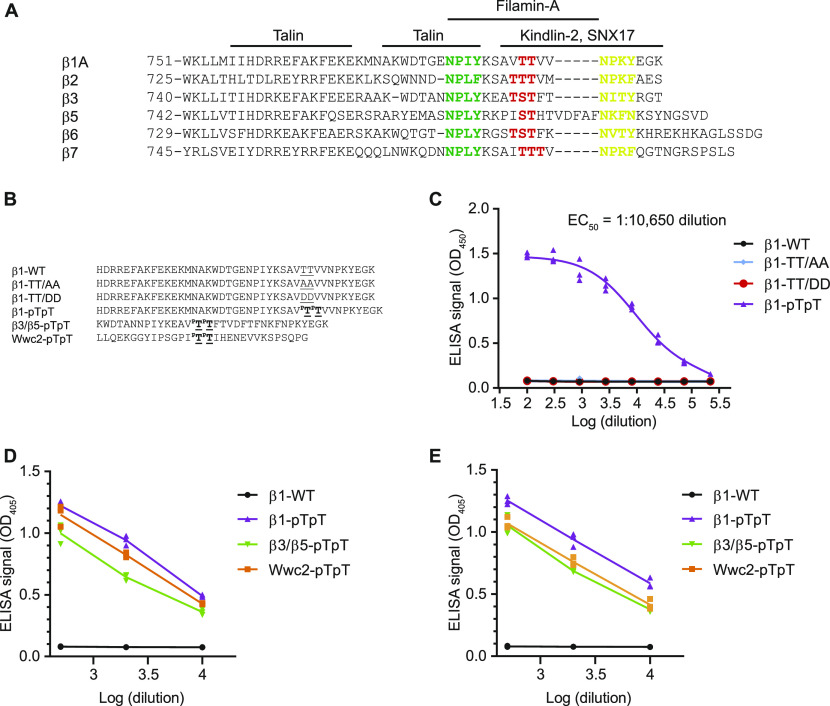
Specificity of the anti–β1-pTpT integrin antibody. **(A)** Alignment of cytoplasmic amino acid residues of mouse integrin β subunits. The conserved threonine motif (red), the proximal NPxY motif (green), the distal NxxY motif (yellow-green), and the binding sites of talin, kindlin-2, SNX17, and filamin-A are indicated. **(B)** Sequences of the synthetic peptides used to determine the specificity of the anti-β1-pTpT antibodies by ELISA. **(C)** Affinity measurement of the anti-β1-pTpT integrin antibody (Thermo Fisher Scientific) to the immobilized β1-pTpT, β1-TT/AA, β1-TT/DD, and β1-WT tail peptides. The dilution factor of the antibody used to generate the titration curve is indicated on the plot. The EC_50_ dilution is indicated. **(D, E)** Quantification of binding of the anti–β1-**p**T**p**T integrin antibody (Thermo Fisher Scientific) (D) and anti–β1-**p**T**p**T integrin antibody (Abcam) (E) towards the indicated β1 integrin tail and phosphorylated Wwc2 peptides. A dilution range between 1:500 and 1:10,000 of the antibodies was used.

Integrins are heterodimeric transmembrane proteins that mediate cell adhesion to extracellular matrix proteins. A feature of integrin function is the requirement of an activation step that is characterized by a reversible shift between unbound, low-affinity (inactive) and bound, high-affinity (active) conformations. Integrin activation is induced and/or maintained by the binding of the FERM (protein 4.1, ezrin, radixin, and moesin) domain-containing adaptor proteins talin and kindlin (Cluzel et al, 2005; Han et al, 2006; Moser et al, 2008; Ye et al, 2013; Theodosiou et al, 2016; Böttcher et al, 2017) to conserved NxxY motifs and adjacent residues in β-integrin cytoplasmic domains (β-tail). The association of the β-tail with talin and kindlin is regulated by signals that control their activation state (termed inside-out signaling). Alternatively, the tyrosine and threonine residues of β-tails (Fig 1A) can be phosphorylated, which alters the affinity for talin and kindlin and the competitive inactivators such as filamin-A and L-plastin (LCP1) (Hynes, 2002; Tseng et al, 2018; Gahmberg et al, 2019). The phosphorylation of the tyrosine residues in the NxxY motifs of the β1-tail is mediated by Src (Hirst et al, 1986; Johansson et al, 1994) resulting in reduced talin and kindlin binding (Anthis et al, 2009; Meves et al, 2011). Interestingly, phosphorylation of either or both tyrosines is dispensable for homeostasis (Chen et al, 2006; Czuchra et al, 2006) but important for enabling anchorage-independent cancer cell growth (Plantefaber & Hynes, 1989; Sakai et al, 2001; Pylayeva & Giancotti, 2006). In the case of the β1-tail threonine phosphorylation, both the identity of the kinase as well as the functional consequences of the phosphorylation event are less investigated and hence, less clear.

Almost all integrin β subunits (T788/T789 in the human and mouse β1 integrin) harbor an evolutionary conserved double-threonine motif, which is located membrane proximally to the NxxY motif that recruits kindlin and filamin-A to β-tails (García-Alvarez et al, 2003; Kiema et al, 2006; Wegener et al, 2007; Moser et al, 2008; Gingras et al, 2009; Böttcher et al, 2012; Liu et al, 2015). The phosphorylation of the double threonine motif in the β1-tail was shown to modulate integrin function, although with conflicting consequences. Structural and biochemical studies suggested that the phosphorylation of the double-threonine motif increases integrin activity (Fagerholm et al, 2005; Takala et al, 2008; Craig et al, 2009; Rehberg et al, 2014; Grimm et al, 2020) by decreasing filamin-A and increasing kindlin binding (Chatterjee et al, 2018; Grimm et al, 2020). On the other hand, the phosphorylation of the double-threonine motif has also been associated with integrin inactivation, adhesion disassembly and the rounding up of mitotic cells (Suzuki & Takahashi, 2003; Kim et al, 2004). The conflicting observations of the β1-T788/T789 phosphorylation for integrin activity are not resolved and the kinase(s) that mediate(s) the β1-T788/T789 phosphorylation have also not been identified. However, the phosphatases PP2A and PPM1F were shown to de-phosphorylate the phospho-threonine motif in β1 integrin tails (Kim et al, 2004; Grimm et al, 2020).

In the present study, we studied the phosphorylation of the double threonine motif in β1-tails in a range of human and mouse cell lines by immunostaining, immunoblotting and mass spectrometry using commercially available anti-β1-pT788/pT789 (β1-pTpT) antibodies. We also determined the consequences of β1-T788/T789 phosphorylation for kindlin and talin binding, cell adhesion and spreading. Our results suggest that the phosphorylation of the β1-T788/T789 motif is, if at all, a rare event that reduces kindlin recruitment to integrins, integrin activity, cell adhesion, and spreading.

## Results

### The β1-T788/T789 phosphorylation in cell lines

Changes in β1-tail phosphorylation during spreading and mitosis have been analyzed with commercially available phosphorylation-specific antibodies (Suzuki & Takahashi, 2003; Kim et al, 2004; Craig et al, 2009; Rehberg et al, 2014; Grimm et al, 2020). To test the specificity of two commercially available anti-β1 integrin pT788/pT789 (β1-pTpT) antibodies we established a direct ELISA using immobilized in-house synthesized β1-tail peptides. The peptides were incubated with different concentrations of the anti-β1-pTpT antibodies to obtain dose–response curves. Whereas the half maximal effective concentration (EC_50_) of the anti-β1-pTpT antibody was reached at a dilution of 1:10,650 for the β1-pTpT peptide, dilutions of the anti-β1-pTpT antibody up to 1:100 bound neither wild-type β1 (β1-WT) nor the phospho-mimicking β1-TT788/789DD (β1-TT/DD) or the non-phosphorylatable β1-TT788/789AA (β1-TT/AA) peptides pointing to a high specificity of the commercial antiserum towards the bi-phosphorylated β1-pTpT cytosolic tails (Fig 1B and C). However, we also detected binding of the two anti-β1-pTpT antibodies to chimeric β3/β5-pTpT peptides and an unrelated peptide with a bi-phosphorylated double-threonine motif derived from the cytosolic scaffolding protein Wwc2 (Höffken et al, 2021) (Fig 1D and E), indicating that the anti-β1-pTpT antibodies are not specific for β1-pT788/pT789 but also react with phosphorylated bi-threonine motifs in β1 integrin-unrelated proteins.

**Figure 2.**
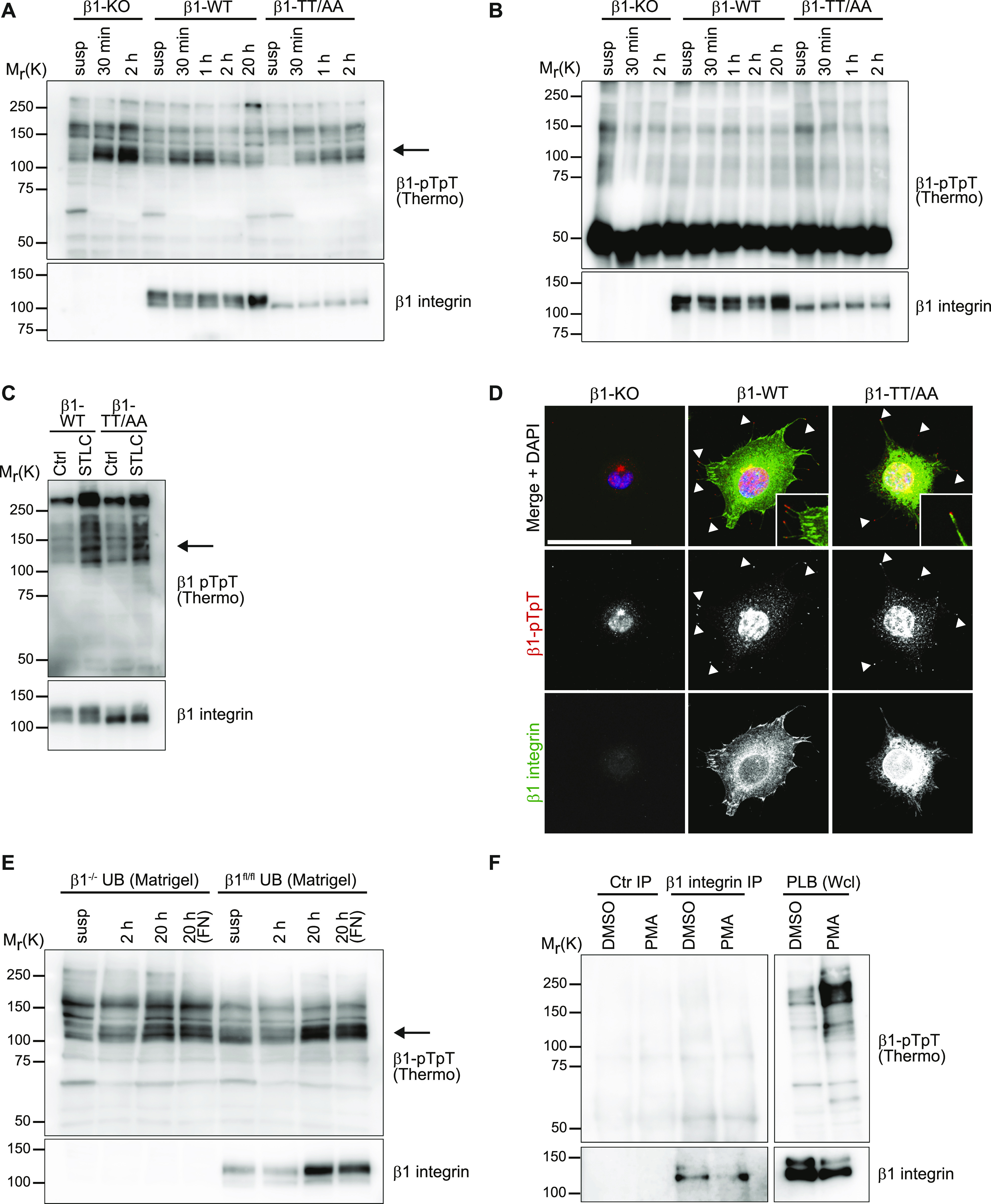
β1 integrin T788/T789 motif phosphorylation is not detectable. **(A, B)** Western blot analysis of whole cell lysates (A) and β1 integrin immunoprecipitations (B) of β1-KO, β1-WT, and β1-TT/AA expressing fibroblasts kept in suspension (susp) or seeded on fibronectin for indicated times. Blots were probed with antibodies against β1-pTpT integrin (Thermo Fisher Scientific) and total β1 integrin. Arrow points toward transiently up-regulated protein with an apparent molecular weight of 125–130 kD. **(C)** Western blots of β1-WT and β1-TT/AA fibroblasts released from 6 h treatment with S-trityl-L-cysteine to enrich for mitotic cells or left untreated (Ctrl) and probed with antibodies against β1-pTpT integrin (Thermo Fisher Scientific) and total β1 integrin. Arrow points toward transiently up-regulated protein with an apparent molecular weight of 125–130 kD. **(D)** Fluorescence microscopy images of β1-KO, β1-WT, and β1-TT/AA expressing fibroblasts seeded for 40 min on fibronectin with antibodies against β1-pTpT integrin (Abcam, red) and total β1 integrin (green). Arrowheads indicate filopodia tips. DAPI was used to stain nuclei. Bar, 20 μm. **(E)** Western blot analysis of wild-type β1 integrin expressing β1^fl/fl^ ureteric bud epithelial cells and β1^−/−^ ureteric bud cells kept in suspension (susp) or seeded on Matrigel for indicated times. Blots were probed with antibodies against β1-pTpT integrin (Thermo Fisher Scientific) and total β1 integrin. Arrow points toward transiently up-regulated protein with an apparent molecular weight of 125–130 kD. **(F)** Western blot analysis of whole cell lysates and β1 integrin immunoprecipitations of PLB-985 (human AML cell line) cells treated for 30 min at 37°C with 100 ng/ml PMA or left unstimulated (DMSO). Blots were probed with antibodies against β1-pTpT integrin and total β1 integrin. Ctr IP, unrelated rabbit antibody.

**Figure S1.**
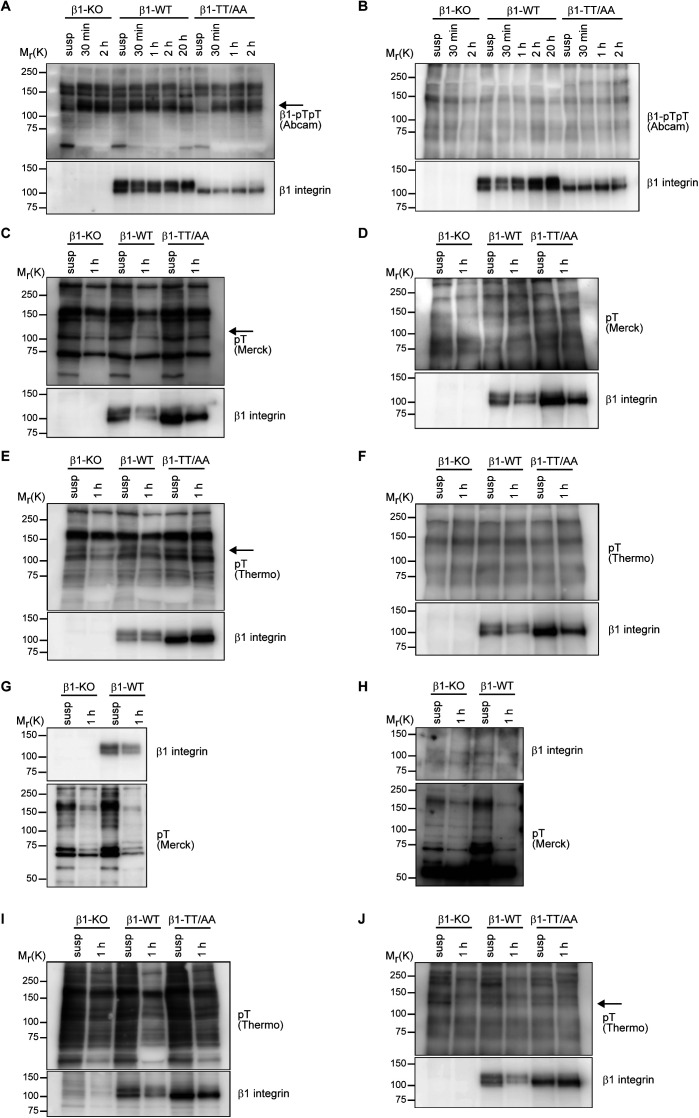
β1 integrin T788/T789 motif phosphorylation is undetectable. **(A, B)** Western blot analysis of whole cell lysates (A) and β1 integrin immunoprecipitates (IPs) (B) of β1-KO, β1-WT, and β1-TT/AA expressing fibroblasts kept in suspension (susp) or seeded for the indicated times on fibronectin (FN). Blots were probed with antibodies against β1-pTpT integrin (Abcam) and total β1 integrin. Arrow points toward transiently up-regulated protein with an apparent molecular weight of 125–130 kD. **(C, D)** Western blot analysis of whole cell lysates (C) and β1 IPs (D) of β1-KO, β1-WT, and β1-TT/AA expressing fibroblasts kept in suspension (susp) or seeded for 1 h on FN. Blots were probed with antibodies against pT (Merck) and total β1 integrin. Arrow points toward proteins with an apparent molecular weight of 125–130 kD. **(E, F)** Western blot analysis of whole cell lysates (E) and β1 IPs (F) of β1-KO, β1-WT, and β1-TT/AA expressing fibroblasts kept in suspension (susp) or seeded for 1 h on FN. Blots were probed with antibodies against pT (Thermo Fisher Scientific) and total β1 integrin. Arrow points toward proteins with an apparent molecular weight of 125–130 kD. **(G, H)** Western blot analysis of whole cell lysates (G) and pT (Merck) generated IPs (H) of β1-KO and β1-WT expressing fibroblasts kept in suspension (susp) or seeded for 1 h on FN. Blots were probed with antibodies against pT and total β1 integrin. **(I, J)** Western blot analysis of whole cell lysates (I) and β1 IPs (J) of β1-KO, β1-WT, and β1-TT/AA expressing fibroblasts kept in suspension (susp) or seeded for 1 h on FN. Cells were treated with 20 μM Calyculin A for 20 min before cell lysis and IP. Blots were probed with antibodies against pT (Thermo Fisher Scientific) and total β1 integrin. Arrow points toward protein with an apparent molecular weight of 125–130 kD.

Next, we analyzed β1-pT788/pT789 levels with the anti-β1-pTpT antibodies in lysates from spread interphase and mitotic mouse fibroblasts seeded on fibronectin (FN). To control the specificity of the immunosignal, we compared β1-pT788/pT789 signals in β1 integrin knockout (β1-KO) fibroblasts, and wild-type β1 integrin (β1-WT) and non-phosphorylatable β1 integrin TT788/789AA (β1-TT/AA) expressing fibroblasts. In line with previous reports (Suzuki & Takahashi, 2003; Grimm et al, 2020), the anti-β1-pTpT antibodies from two different commercial sources detected a transiently up-regulated protein with an apparent molecular weight of 125–130 kD, which corresponds to the apparent molecular weight of the mature β1 subunit, in interphase fibroblasts seeded on FN or fibroblasts stalled in mitosis (Figs 2A and C and S1A). However, signals with same size and kinetics were also detected in β1-KO and β1-TT/AA-expressing fibroblasts, suggesting that the anti-β1-pTpT antibodies recognize β1 integrin-unrelated protein(s). Moreover, the 125–130 kD signal was undetectable by anti-β1-pTpT immunoblotting of β1 integrin immunoprecipitates (IPs) obtained with a polyclonal rabbit anti-mouse β1 integrin antiserum raised against the β1 integrin ectodomain (Figs 2B and S1B), which further corroborates the notion that the commercial anti-β1-pTpT antibodies do not detect the phosphorylated β1-T788/T789 motif in FN-seeded fibroblasts in immunoblots. To further validate β1-tail threonine phosphorylation, we generated lysates from FN-seeded fibroblasts and performed immunoblotting with two commercial anti-phospho-threonine (anti-pT) antibodies. These antibodies should recognize all phosphorylated threonine residues, irrespective of the protein backbone. The anti-pT antibodies detected proteins with a molecular weight of 125–130 kD in β1-WT- as well as in β1-KO- and β1-TT/AA–expressing fibroblasts (Fig S1C and E). These unspecific signals were not detectable anymore with the anti-pT antibodies in immunoblots of IPs produced with the polyclonal anti-mouse β1 integrin antiserum from β1-WT-, β1-KO-, and β1-TT/AA-expressing cells (Fig S1D and F). Conversely, the polyclonal anti-mouse β1 integrin antiserum was also not able to detect β1 integrin signals in IPs produced with the anti-pT antibodies (Fig S1G and H and data not shown). Finally, we immunoblotted IPs from FN-seeded cells pre-treated with the PP2A inhibitor Calyculin A. The anti-pT antibody (Thermo Fisher Scientific) detected a band of 125–130 kD in anti-β1 integrin IPs. However, this band was present in both, β1-WT and β1-KO cells (Fig S1I and J). Together, these data indicate that under our experimental conditions β1-T788/T789 phosphorylation is either not occurring or not detectable.

**Figure S2.**
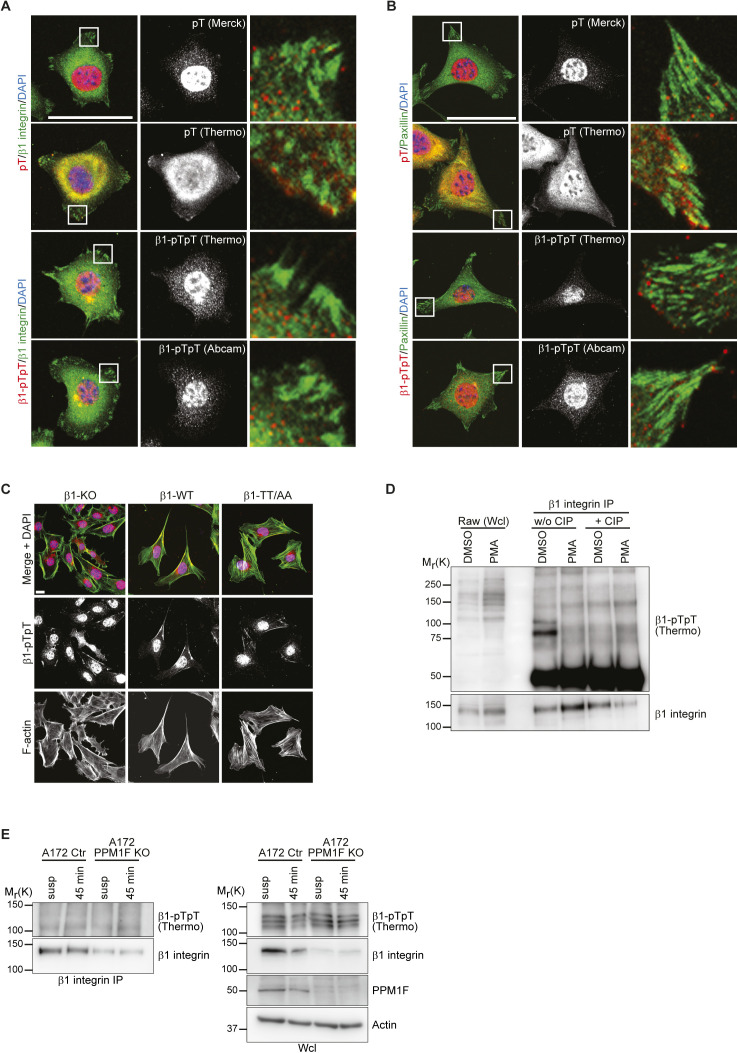
β1 integrin T788/T789 motif phosphorylation is not detectable by immunofluorescence and by Western blotting. **(A)** Fluorescence images of β1-WT expressing fibroblasts allowed to spread for 40 min on fibronectin (FN) with antibodies against β1-pTpT integrin, phospho-threonine (pT, red), or total β1 integrin (green). DAPI was used to stain nuclei. Bar, 50 μm. **(B)** Fluorescence images of β1-WT expressing fibroblasts allowed to spread for 2 h on FN with antibodies against β1-pTpT integrin, pT (red) or paxillin (green). DAPI was used to stain nuclei. Bar, 50 μm. **(C)** Fluorescence microscopy images of β1-KO, β1-WT, and β1-TT/AA expressing fibroblasts seeded for 4 h on FN with antibodies against β1-pTpT integrin (Abcam, red) and phalloidin-Alexa 488 (F-actin, green). DAPI was used to stain nuclei. Bar, 20 μm. **(D)** Western blot analysis of whole cell lysates and β1 integrin immunoprecipitations of RAW 264.7 cells treated for 30 min at 37°C with 100 ng/ml PMA or left unstimulated (DMSO). IP samples were treated with calf intestinal phosphatase for 30 min at 37°C or left untreated. Blots were probed with antibodies against β1-pTpT integrin (Thermo Fisher Scientific) and total β1 integrin. **(E)** Western blot analysis of β1 integrin IPs (left) or whole cell lysates (right) of A172 control or A172 PPM1F KO cells kept in suspension (susp) or seeded 45 min at 37°C on FN. Blots were probed with antibodies against β1-pTpT integrin (Thermo Fisher Scientific), total β1 integrin, PPM1F, and actin.

The immunostaining of β1-WT expressing fibroblasts seeded for 40 min or 2 h on FN with anti-β1-pTpT and anti-pT antibodies produced signals in the nucleus and cytosol, but not in β1 integrin-positive FAs of β1-WT–expressing fibroblasts. One anti-β1-pTpT antibody (Abcam) produced signals at filopodia tips and along actin fibers in β1-WT– as well as in β1-KO– and β1-TT/AA–expressing fibroblasts (Figs 2D and S2A–C).

**Figure S3.**
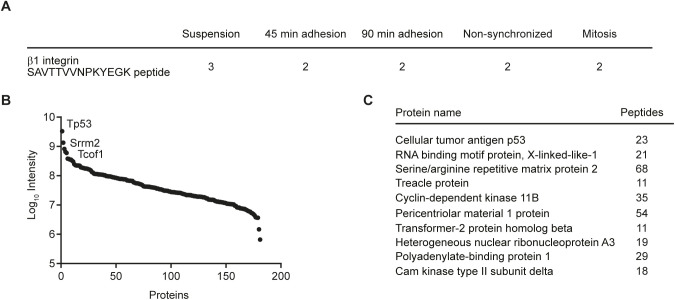
Proteomic analysis of β1 integrin T788/T789 motif phosphorylation. **(A)** Number of β1-T788/T789–containing peptides identified by tandem mass spectrometry in β1-WT fibroblasts fibroblasts kept in suspension, seeded for the indicated times on fibronectin or treated with S-trityl-L-cysteine to enrich for mitotic cells or left untreated (non-synchronized) after immunoprecipitation of β1 integrin. β1 integrin T788/T789 phosphorylation was not detected. **(B)** Intensity dynamic range of 181 quantified proteins by tandem mass spectrometry after β1-pTpT immunoprecipitations from β1-WT fibroblasts. Proteins with more than three unique peptides and at least threefold higher abundance in β1-pTpT samples compared with control IgG antibody samples were listed. **(C)** List of top 10 proteins with highest intensity values.

We also performed mass spectrometry to identify β1-pT788/pT789 phosphorylation in β1 integrin IPs obtained from β1-WT interphase fibroblasts either kept in suspension or seeded for 45 and 90 min on FN, or β1-WT fibroblasts arrested in mitosis with polyclonal rabbit anti-mouse β1 integrin antiserum. Whereas the non-phosphorylated β1-T788/T789–containing peptide was clearly detectable in the IPs, the phosphorylated β1-pT788/pT789 peptides were neither found in β1-WT interphase nor in mitotically arrested fibroblasts (Fig S3A). Also the enrichment of β1-pT788/pT789 integrin in FN-seeded β1-WT fibroblasts by immunoprecipitation with anti-β1-pTpT antibodies followed by tandem mass spectrometry (MS/MS) revealed that β1-T788/T789-containing peptides were not among the 181 proteins that were minimally threefold enriched in β1-pTpT IPs over control IgG IPs (Fig S3B and C).

To confirm our findings also in epithelial and hematopoietic cells, we immunoblotted whole cell lysates from mouse ureteric bud (UB) epithelial cells, mouse macrophage line RAW 264.7 and the acute myeloid leukemia cell line PBL-985 with the commercial anti-β1-pTpT antibodies. The UB cells were established from UBs isolated from mouse embryos carrying floxed *Itgb1* alleles (β1^fl/fl^UB) and subsequently transduced with *Cre* to generate β1^−/−^UB cells. The endogenous wild-type β1 integrin expressing β1^fl/fl^UB and β1^−/−^UB cells seeded for different times on Matrigel displayed a similar transient increase in an apparent molecular weight protein of 125–130 kD with the anti-β1-pTpT antibodies as observed in fibroblasts, indicating that the immunosignal produced by the anti-β1-pTpT antibodies is also unspecific in the UB epithelial cells (Fig 2E). The RAW 264.7 and PLB-985 cell lines were analyzed before and after stimulation with phorbol 12-myristate 13-acetate (PMA), which was shown to induce the phosphorylation of the threonine motif in the β2-tail (Fagerholm et al, 2002; Hilden et al, 2003; Nurmi et al, 2006; Takala et al, 2008). Although the PMA treatment increased the signal intensity of multiple protein bands that were detected with the anti-β1-pTpT integrin antibody, none were detected in β1 integrin IPs indicating that β1-T788/T789 phosphorylation is, if it was induced at all, below the detection limit of these commercial anti–β1-pTpT antibodies (Figs 2F and S2D).

To rule out that high levels of the PPM1F phosphatase cause the loss of β1-T788/T789 phosphorylation, we analyzed the β1-T788/T789 phosphorylation in A172 PPM1F knockout cells (Grimm et al, 2020). The experiments revealed comparable anti-β1-pTpT signals in Western blots of suspended or FN-seeded WT and PPM1F-deficient A172 cells. Furthermore, these immunosignals were undetectable in β1 integrin IPs (Fig S2E), which altogether indicates that β1-T788/T789 phosphorylation is either below the detection limit or absent in all cell lines tested.

### The phosphorylated integrin β1-T788/T789 motif fails to bind kindlin and SNX17

**Figure 3.**
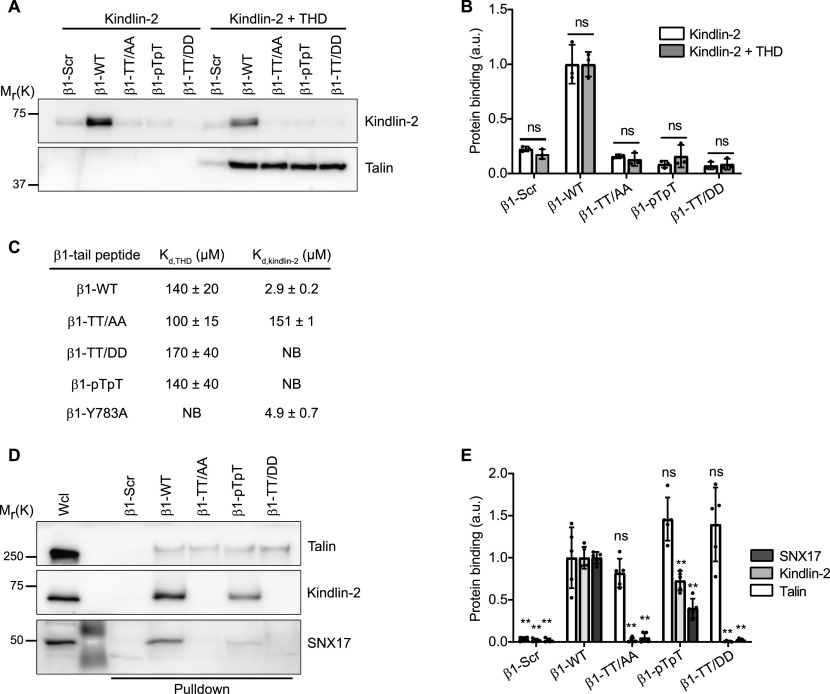
β1 integrin T788/T789 phosphorylation curbs kindlin-2 and SNX17 but not talin binding. **(A)** Streptavidin-bead pull-down assay with the indicated biotinylated β1-integrin cytoplasmic tail (β1-tail) peptides and recombinant kindlin-2, or a combination of kindlin-2 and talin head domain. After pull-down, bound proteins were detected by Western blotting. **(B)** Densitometric analysis of kindlin-2 binding to indicated β1-tail peptides in the absence and presence of talin head domain. Data are mean ± SD. *n* = 3. ns, not significant (Mann–Whitney *U* test). **(C)** Affinities of talin and kindlin-2 for indicated Atto488-labeled β1-tail peptides determined by microscale thermophoresis. **(D)** Indicated biotinylated β1-tail peptides were analyzed for binding to endogenous talin, kindlin-2, and SNX17 by Western blotting. Wcl, whole cell lysate. **(E)** Densitometric analysis of talin, kindlin-2, and SNX17 to the indicated β1-tail peptides. Data are mean ± SD of *n* = 5 independent experiments. **P < 0.01; ns, not significant (Mann–Whitney *U* test). Statistical significances are calculated between β1-WT and mutant β1-tail peptides.

The T788/T789 motif of the β1-tail is involved in binding several cytoplasmic proteins including kindlin, SNX17, and filamin-A (Fig 1A). To explore the consequences of β1-T788/T789 phosphorylation for kindlin-2 binding, we tested whether synthetic peptides representing the cytoplasmic domains of β1-WT, β1-TT/AA, β1-pTpT, β1-TT/DD, or scrambled peptides are able to pull down recombinant kindlin-2 and talin-1 head domain (THD). Expectedly, the THD was pulled down with all β1-tail peptides (Fig 3A and B). In contrast, recombinant kindlin-2 was only pulled down with β1-WT but not with β1-TT/AA, β1-TT/DD, and β1-pTpT, irrespective whether THD was absent or present in the pull-down assays (Fig 3A and B). Whereas affinity measurements using microscale thermophoresis (MST) revealed similar affinities of THD for phospho-inhibitory, phospho-mimicking, or phosphorylated β1-T788/T789 tail peptides as for β1-WT tails, the affinity of kindlin-2 for β1-TT/AA was strongly reduced and for β1-TT/DD and β1-pTpT not measurable anymore (Fig 3C). Expectedly, disruption of the membrane-proximal NPxY motif (β1-Y783A) abolished THD binding without influencing the affinity of kindlin-2 (Fig 3C).

Pull-down experiments of whole cell lysate showed that talin efficiently bound to all β1-tail peptides tested, whereas kindlin-2 and SNX17 readily bound to β1-WT, their binding to the β1-pTpT peptide was reduced to 27 ± 11% and 60 ± 11% (mean ± SD, *n* = 5), respectively, and lost to β1-TT/AA, β1-TT/DD, and the scrambled peptides (Fig 3D and E). Interestingly, mass spectrometry-based measurements of the phosphorylation state of the β1-pTpT peptide revealed that their incubation in whole cell lysates reduced their phosphorylation by around 40%, despite the presence of phosphatase inhibitors (see the Materials and Methods section), which explains the decreased binding to kindlin-2 and SNX17. Altogether these findings suggest that phosphorylation of the double-threonine motif in β1-T788/T789 blocks kindlin-2 and SNX17 binding to β1 integrins.

### β1-TT/DD and β1-TT/EE substitutions curb integrin activation and ligand binding

**Figure 4.**
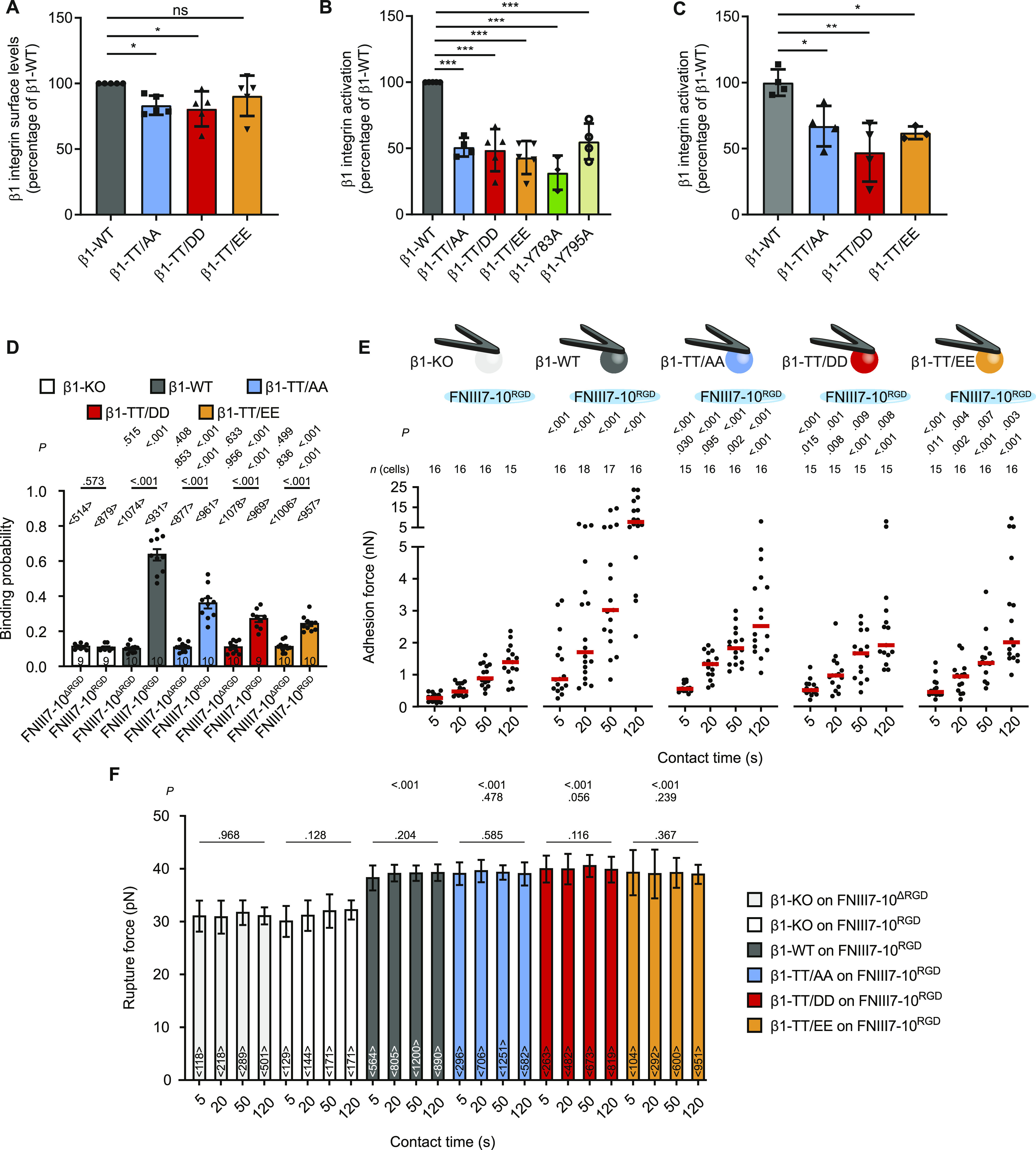
Substitutions of threonine residues in the β1 integrin cytoplasmic domain reduce integrin activation and functions. **(A)** Quantification of cell surface expression of β1 integrin on β1-KO fibroblasts and β1 integrin re-expressing fibroblasts measured by flow cytometry. The integrin levels were normalized to the levels on WT fibroblasts. Data are mean ± SD. *n* = 5. *P < 0.05; ns, not significant (one-way ANOVA with Kruskal–Wallis test). Statistical significances are calculated between β1-WT and mutant cell lines. **(B)** Quantification of β1 integrin activation on the indicated fibroblast cell lines measured by 9EG7 binding by flow cytometry analysis and corrected for total β1 integrin levels. Data are mean ± SD. *n* = 3–5. ***P < 0.001 (one-way ANOVA). Statistical significances are calculated between β1-WT and mutant cell lines. **(C)** Quantification of active β1 integrin (9EG7) and total β1 integrin levels by immunostaining of the indicated fibroblast cell lines after seeding on fibronectin-coated dishes for 40 min. Data are mean ± SD. *n* = 4 (*n* = 3 for TT/EE). **P < 0.01; *P < 0.05 (one-way ANOVA). Statistical significances are calculated between β1-WT and mutant cell lines. **(D)** Binding probabilities of the indicated fibroblast cell lines quantified by single-cell force spectroscopy after contact with FNIII7-10^RGD^ or FNIII7-10^ΔRGD^ for ∼300 ms at a contact force of 200 pN. Binding probabilities were calculated as ratio of force–distance curves showing unbinding events and total attempts. Dots represent binding probabilities of single fibroblasts, bars the mean and the error bars the SEM. 9–10 cells analyzed. Top row of *P*-values compare the given condition with β1-KO fibroblasts on the respective substrate, lower row of *P*-values compare given condition with β1-KO fibroblasts re-expressing β1-WT. *P*-values on bars compare binding probabilities of given cell line to FNIII7-10^RGD^ and FNIII7-10^ΔRGD^. **(E)** Adhesion forces of single fibroblasts to FNIII7-10^RGD^ after given contact times. Dots represent adhesion forces of single fibroblasts (*n* = 15–18), red bars their mean. Top row of *P*-values compares adhesion forces of given fibroblast line to adhesion forces of β1-KO fibroblasts and lower row of *P*-values to adhesion forces to β1 WT fibroblasts. **(F)** Rupture forces of single unbinding events were analyzed from force–distance curves of adhesion force experiments, data taken from (E) and Fig S5G. Bars display the median rupture force for the given cell line and contact time, the error bars the 95% confidence interval of the median and <*n*> the number of rupture events analyzed. *P*-values on bars show statistically significant differences between rupture forces after different contact times for given cell line and condition. *P*-values compare rupture forces of given condition with rupture forces of β1-KO fibroblasts (top row) or β1-WT fibroblasts (bottom row) on FNIII7-10^RGD^.

**Figure S4.**
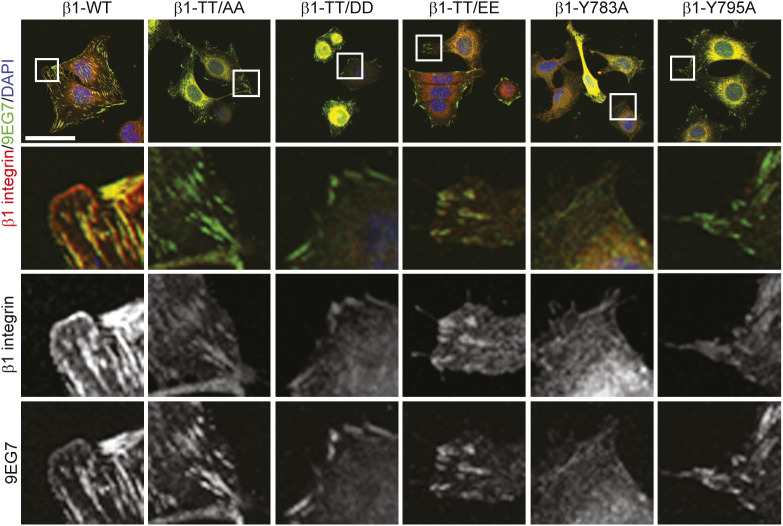
Severe β1 integrin activation defects in fibroblasts expressing β1 integrin TT/DD and TT/EE. Confocal fluorescence images of fibronectin-seeded β1-deficient (β1-KO) fibroblasts re-expressing either β1-WT, β1-TT/AA, β1-TT/DD, β1-TT/EE, β1-Y783A, or β1-Y795A integrins stained with antibodies against active β1 integrin (9EG7, green) and total β1 integrin (red). DAPI was used to stain nuclei. Bar, 20 μm.

To investigate the potential role of the phosphorylation of β1-T788/T789 for β1 integrin activation and function in cells, we re-expressed comparable levels of β1-TT/DD and β1-TT/EE, or β1-TT/AA in β1 integrin-deficient fibroblasts (β1-KO). In addition, we expressed β1-Y783A, which disrupts the membrane-proximal NPxY motif required for talin and filamin-A binding, and β1-Y795A, which disrupts the membrane-distal NxxY motif required for kindlin and SNX17 binding. Flow cytometry (FC) measurements and immunostainings revealed reduced levels of the conformation-specific β1 integrin activation epitope 9EG7 on the surface or in FAs of FN-seeded β1-KO fibroblasts reconstituted with β1-Y795A, β1-TT/AA, β1-TT/DD, and β1-TT/EE (Figs 4A–C and S4), indicating that the double-threonine substitution with either phospho-mimicking or phospho-inhibitory residues reduces integrin activity in fibroblasts.

**Figure S5.**
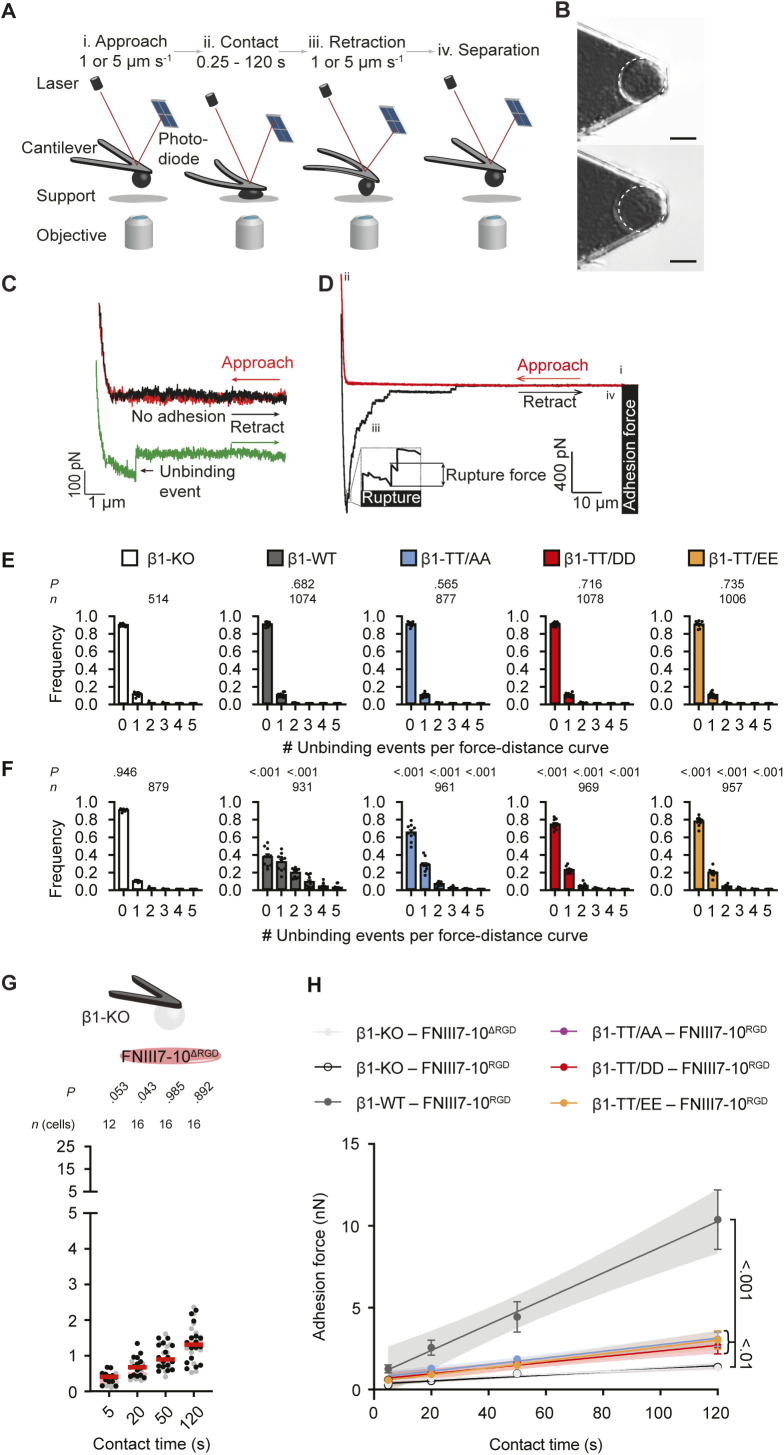
Atomic force microscopy-based single-cell force spectroscopy setup to quantify adhesion properties of fibroblasts to different full-length fibronectin and FNIII7-10 fragments. **(A)** Single-cell force spectroscopy setup. (i) Single fibroblasts are incubated for ∼5–15 min on a concanavalin A-coated cantilever to assure firm attachment. (ii) Cantilever-attached fibroblasts are approached to fibronectin fragment (FNIII7-10)–coated supports. (iii, iv) After 0–120 s contact time, the cantilever-bound fibroblast is retracted vertically until the fibroblast is fully detached from substrate to either quantify binding probabilities or measure adhesion force between fibroblast and support. During adhesion experiments, cantilever deflections are recorded and displayed in force–distance (FD) curves. **(B)** (top) A single round fibroblast attached to the cantilever tip used to measure adhesion forces until (bottom) the fibroblast’s morphology changed (i.e., spreading). **(C)** FD curves representing binding of single integrins on fibroblasts to FNIII7-10-coated substrate. To record single integrin unbinding events, fibroblasts are approached to the substrate (red curve) until they contact the substrate at minimal contact force (200 pN) and contact time (∼300 ms) and are then retracted (black/green curve). The green FD curve shows a single adhesion (unbinding) event, whereas the black FD curve shows no adhesion event. **(D)** A representative FD curve after longer contact times (5–120 s) shows different features: the retraction FD curve (black) records adhesion forces of the fibroblast, which represent the maximum downward deflection of the cantilever and thus, the force required to separate the fibroblast from the substrate. During detachment of the fibroblast from the substrate, single receptor unbinding events are observed (ruptures). Ruptures are recorded when bonds formed between integrins that remain associated with the cell cortex and substrate fail. Tether events (longer plateaus) are recorded when a membrane tether extrudes from the cell body with a single or multiple integrins at its tip and occur when the integrin linkage to the actomyosin cytoskeleton is either too weak to resist the mechanical load applied or non-existent. **(E, F)** Probability of multiple unbinding events per FD curve in binding probability experiments of given fibroblast lines to (E) FNIII7-10^ΔRGD^ or (F) FNIII7-10^RGD^ (data taken from Fig 4E). Data are mean + SEM and dots represent probabilities of single fibroblasts. In (E) *P*-values compare the distribution of unbinding events of given condition to β1-KO fibroblasts. In (F) left *P*-values compare the unbinding event distributions of the given cell line to FNIII7-10^RGD^ with unbinding event distributions of given cell line to FNIII7-10^ΔRGD^, the middle *P*-value with β1-KO fibroblasts to FNIII7-10^RGD^ and the right *P*-values with β1-WT fibroblasts on FNIII7-10^RGD^. *n* gives the number of force distance-curves analyzed per condition. *n*(cells) = 9–10. **(G)** Adhesion forces of single β1-KO fibroblasts to FNIII7-10^ΔRGD^ after given contact times. Dots represent adhesion forces of single fibroblasts, red bars their mean. In grey adhesion forces of β1 KO fibroblasts to FNIII7-10^RGD^ are given as reference. *n* = 12(16). *P*-values compare adhesion forces of given and reference. *P*-values were calculated by two sided Mann–Whitney *U* tests. **(H)** Adhesion strengthening for each fibroblast line over time. Dots represent mean and bars SEM. Data taken from Fig 4E.

Next, we investigated the consequences of altered β1 integrin activity by characterizing the binding probabilities of β1-KO fibroblasts reconstituted with β1-WT, β1-TT/AA, β1-TT/DD, and β1-TT/EE by atomic force microscopy (AFM)-based single-cell force spectroscopy (SCFS) at molecular resolution (Fig S5A–D). We attached single fibroblasts to concanavalin A–coated cantilevers, approached them to FN fragments containing or lacking the RGD motif (FNIII7-10^RGD^ or FNIII7-10^ΔRGD^) until a contact force of ∼200 pN was reached, maintained the cantilever at a constant height for 250 ms, and subsequently retracted the fibroblast from the substrate. This resulted in a total contact time of ∼300 ms, which is sufficient for α5β1 integrins to bind ligand (Benito-Jardón et al, 2020). We blocked the contribution of αv-class integrins to the binding probability by adding 1 μM cilengitide to the medium. All tested fibroblast cell lines showed similar background binding probabilities to integrin-binding deficient FNIII7-10^ΔRGD^. Whereas cilengitide-treated β1-KO fibroblasts also showed background binding probability to FNIII7-10^RGD^, cilengitide-treated β1-KO fibroblast reconstituted with β1-TT/AA, β1-TT/DD, and β1-TT/EE exhibited reduced integrin-specific binding probabilities to FNIII7-10^RGD^ compared with β1-WT expressing fibroblasts (Fig 4D). The β1-WT, β1-TT/AA, β1-TT/DD, and β1-TT/EE expressing fibroblasts showed more than one unbinding event in <3% of binding probability experiments on FNIII7-10^ΔRGD^, whereas β1-WT fibroblasts exhibited multiple unbinding events (up to five) FNIII7-10^RGD^ in 32.3 ± 19.5% (mean ± SD, *n* = 9 cells), which indicates that ∼300 ms is sufficient for multiple integrins to bind ligand (Fig S5E and F). In line with reduced binding probability, all the β1 integrins carrying TT-substitutions reduced the probability of multiple integrin binding within the chosen contact time (β1-TT/AA: 7.7 ± 4.0%; β1-TT/EE: 4.6 ± 4.1%; β1-TT/DD: 3.8 ± 3.2%; all *P* < 0.001 and *n* = 10 cells). Altogether, the SCFS results show that the phospho-inhibitory β1-TT/AA as well as the phospho-mimicking β1-TT/DD or β1-TT/EE substitutions drastically reduce the binding rate of β1-class integrins to FN.

### β1-TT/DD and β1-TT/EE substitutions reduce cell adhesion and spreading

Next, we investigated the adhesion strengthening of β1-KO fibroblasts expressing β1-WT, β1-TT/AA, β1-TT/DD, and β1-TT/EE to FN in the presence of cilengitide by quantifying their adhesion forces to FNIII7-10^RGD^ at contact times ranging from 5 to 120 s (Figs 4E and S5G). In line with previous reports (Bharadwaj et al, 2017; Benito-Jardón et al, 2020), fibroblasts expressing β1-WT integrins rapidly strengthened adhesion, which was quantified as the slope of a linear fit through all adhesion forces obtained from indicated contact times (Fig S5H). In contrast, fibroblasts expressing β1-TT/AA, β1-TT/DD, and β1-TT/EE established markedly reduced adhesion forces and adhesion strengthening to FNIII7-10^RGD^. To determine the impact of the TT substitutions on the maximum force single β1-integrins can bear before they detached from their ligands, we quantified single-rupture forces from force distance (FD) curves (Fig 4F). Whereas β1-KO fibroblasts showed non-specific single-rupture forces from FNIII7-10^RGD^ and FNIII7-10^ΔRGD^ at all contact times, β1-WT, β1-TT/AA, β1-TT/DD, and β1-TT/EE expressing fibroblasts exhibited similar single-rupture forces from FNIII7-10^RGD^ that were contact time-independent and higher compared with β1-KO fibroblasts. These results show that single FN-bound β1-integrins can bear similar forces irrespective of the TT-substitution, whereas their ability to strengthen adhesion is dramatically reduced.

**Figure 5.**
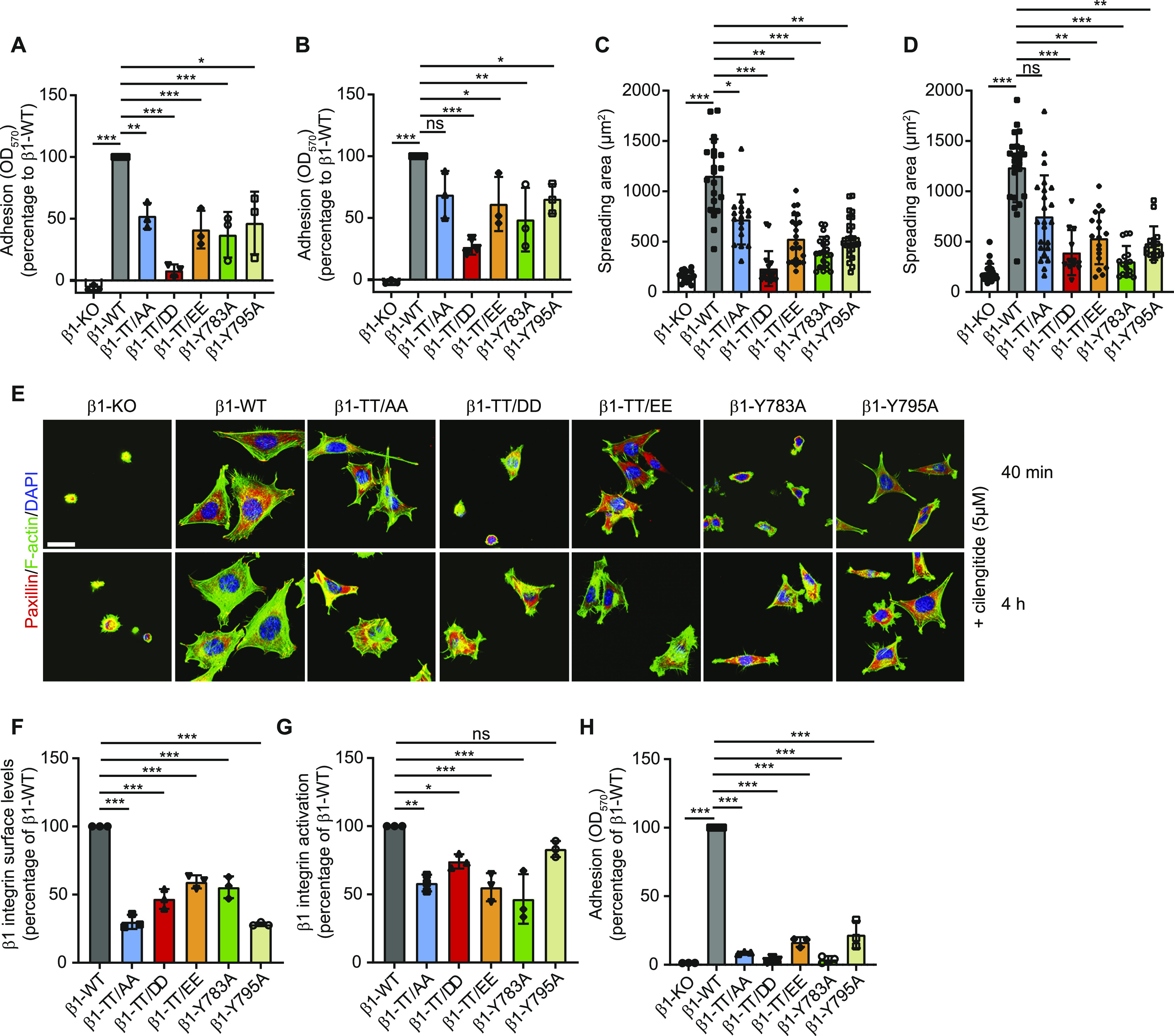
β1 integrin TT/DD and TT/EE inhibit cell adhesion and spreading. **(A, B)** Quantification of cell adhesion on fibronectin (FN) 15 min (A) and 40 min (B) after seeding serum-starved fibroblasts in the presence of 5 μM cilengitide. Data are mean ± SD. *n* = 3. ***P < 0.001; **P < 0.01; *P < 0.05; ns, not significant (Repeated measures [RM] one-way ANOVA). Statistical significances are calculated between β1-WT and mutant cell lines. **(C, D)** Quantification of the cell area after spreading on FN for 40 min (C) and 4 h (D) in the presence of 5 μM cilengitide. Data are mean ± SD of *n* > 30 cells. ***P < 0.001; **P < 0.01; *P < 0.05; ns, not significant (one-way ANOVA with Kruskal–Wallis test). Statistical significances are calculated between β1-WT and mutant cell lines. **(E)** Immunostaining of paxillin (red) and F-actin (green) in fibroblasts expressing indicated β1 integrin mutants and seeded on FN-coated coverslips for 40 min or 4 h in the presence of 5 μM cilengitide before immunostaining. DAPI was used to stain nuclei. Scale bar, 20 μm. **(F)** Quantification of cell surface expression of β1 integrin on β1^−/−^ureteric bud cells re-expressing β1-WT integrin or the indicated β1 integrin tail mutants measured by flow cytometry. The integrin levels were normalized to the levels on β1-WT cells. Data are mean ± SD. *n* = 3. ***P < 0.001 (RM one-way ANOVA). Statistical significances are calculated between β1-WT and mutant cell lines. **(G)** Quantification of integrin activation after staining total and active β1 integrin (9EG7) by flow cytometry. The mean fluorescence intensity ratio of active to total β1 integrin was calculated and normalized to the β1-WT sample. Data are mean ± SD. *n* = 3. ***P < 0.001; **P < 0.01 (RM one-way ANOVA). Significances are calculated between β1-WT and mutant UB cell lines. **(H)** Quantification of cell adhesion on Matrigel 30 min after seeding. Data are mean ± SD. *n* = 3. ***P < 0.001 (RM one-way ANOVA). Statistical significances are calculated between β1-WT and mutant UB cell lines.

Plate-and-wash assays on FN in the presence of cilengitide revealed diminished adhesion of β1-TT/AA, β1-TT/DD, and β1-TT/EE expressing fibroblast to FN 15 min and to a lesser extent 45 min after seeding when compared with β1-WT fibroblasts, which was most pronounced for the β1-TT/DD-expressing fibroblasts (Fig 5A and B). Spreading of β1-TT/AA, β1-TT/DD, and β1-TT/EE expressing fibroblast on FN for 40 min or 4 h in the presence of cilengitide was also significantly impaired compared with β1-WT cells (Fig 5C–E) with the strongest defect in β1-TT/DD fibroblasts.

To confirm these findings in epithelial cells, we generated β1 integrin-deficient mouse β1^−/−^UB cells rescued with β1-WT, β1-TT/AA, β1-TT/DD, β1-TT/EE, and the talin- and kindlin-binding–deficient β1-Y783A and β1-Y795A, respectively. Irrespective of the integrin tail mutation, the UB cells displayed reduced total β1 integrin and reduced 9EG7 epitope levels on their surface (Fig 5F and G). Furthermore, all β1-tail mutant expressing UB cell lines almost completely failed to adhere to Matrigel (Fig 5H). Thus, the adhesion inhibiting effect of the β1-TT/AA, β1-TT/DD, and β1-TT/EE mutations is not limited to fibroblasts but is also observed in epithelial cells, which altogether indicates that phospho-mimicking as well as phospho-inhibiting substitutions of the β1-T788/T789 curb integrin activation, cell adhesion and spreading.

## Discussion

In the present article, we investigated the role of the conserved integrin threonine motif in the β1-tail for integrin activation and function. We report that β1-integrin phosphorylation at T788/T789 is below detection level in five cell lines analyzed, in vitro phosphorylated β1-T788/T789 tail peptides fail to bind kindlin-2, and phospho-mimicking β1-TT788/789DD integrin expressing cells show reduced β1 integrin activation, adhesion and spreading.

The phosphorylation of the conserved double-threonine motif in integrin β1 tails has been investigated in several laboratories using commercial antibodies that were raised in rabbits against a chemically synthesized phosphopeptide derived from the β1 integrin tail region containing pT788/pT789 (Suzuki & Takahashi, 2003; Kim et al, 2004; Bhalla et al, 2009; Craig et al, 2009; Rehberg et al, 2014; Grimm et al, 2020). We established a sensitive ELISA assay, which confirmed that two commercial anti-β1-pTpT antibodies bind to synthetic bi-phosphorylated β1-pTpT cytoplasmic tail peptides. However, this assay also showed binding of the antibodies to different integrin β-subunits and an unrelated bi-phospho-threonine containing peptide. In line with the binding to phosphorylated double-threonine motifs unrelated to the β1-tail, the anti-β1-pTpT antibodies also detected protein(s) with an apparent molecular weight of 120–130 kD in cell lysates, which—although corresponding to the molecular weight range of the mature β1-integrin subunit—was also visible in β1 integrin-deficient cells. Our mass spectrometry analysis of anti-β1-pTpT IPs did not detect β1 integrin tail peptides. Interestingly, however, we identified several proteins with an apparent molecular weight of 120–130 kD, including Wwc2, Tcof1, and Larp1, which could be the source of the unspecific band. These findings strongly suggest that the anti-β1-pTpT antibodies are not specific for β1-pT788/pT789 integrin but rather detect unrelated proteins containing bi-phosphorylated threonine residues, which probably served as major immune-epitope during rabbit immunization. Because none of the studies that used the anti-β1-pTpT antibodies controlled the specificity of their results with β1 integrin-deficient cells off-target signals may have been misinterpreted.

Western blotting, mass spectrometry and immunostaining failed to detect the phosphorylation of the double threonine motif in β1-T788/T789 integrin in several cell lines. This observation indicates that the phosphorylation of the double-threonine motif in β1-tails is below the detection limit of our assays, or only existent in cell types that have not been analyzed in our study. Our conclusion that the phosphorylation of the double-threonine motif in β1-tails is, if at all, a rare event gains support from unbiased high-throughput mass spectrometry data of numerous cell types and experimental conditions. The in silico analysis of these data source revealed that the phosphorylation of β1-T788/T789 was not identified so far and two studies reported the phosphorylation of the single threonine-789 in β1-tails (Shiromizu et al, 2013; Bian et al, 2014). In contrast, phosphorylation of tyrosine-783 and tyrosine-795 in β1 integrin tails has been reported in hundreds of independent studies (phosphosite.org).

Our study also revealed that expression of phospho-mimicking β1-TT788/789DD or β1-TT788/789EE integrins in β1 integrin-null fibroblasts reduced β1 integrin activation, integrin–FN binding, adhesion strengthening, and cell spreading, whereas binding strength of single integrins to FN was unaffected by the threonine substitutions. These phenotypes are likely due to the reduced kindlin-2 affinity to β1-tails, which was clearly evident in pull-down and MST experiments with β1-TT788/789DD as well as in vitro phosphorylated β1-pT788/pT789 tail polypeptides, irrespective whether talin was present or absent during the measurements. Interestingly, the severity of the cellular phenotypes of wild-type and mutant β1 integrin-expressing cells perfectly correlated with the reduced kindlin affinities measured by MST. Whereas the affinity of kindlin-2 for β1-TT788/789AA tail peptides was reduced and β1-TT788/789AA–expressing fibroblasts showed reduced cell adhesion and spreading, β1-TT788/789DD substitutions abolished kindlin-2 binding and more strongly reduced cell adhesion and spreading.

Our data are in contrast to previous studies which suggested that the phosphorylation of the double-threonine motif through Ndr2 or after PPM1F depletion increases integrin activity (Rehberg et al, 2014; Grimm et al, 2020). Unfortunately, neither of the two studies analyzed the effects of β1-T788/T789 phosphorylation in cells expressing phospho-mimicking β1-TT788/789 variants. It is also unfortunate that the two studies did not include β1 integrin-deficient cells to control for the detection of integrin-unrelated protein(s) accidently recognized by the anti-β1-pTpT antibodies. We do not challenge that Ndr2 (Rehberg et al, 2014) and PPM1F (Grimm et al, 2020) alter integrin activity. However, we strongly assume, based on our findings, that Ndr2 and PPM1F alter integrin activity by modifying targets other than β1-T788/T789.

In conclusion, our findings challenge the existence of a β1-T788/T789 phosphorylation switch as a major mechanism used by cells to regulate integrin activity and function. However, if such an activity switch would exist, the phosphorylation of the threonine motif in the β1-tail would inhibit rather than promote kindlin-2 binding, integrin activity and function.

## Materials and Methods

### Antibodies

The following antibodies or molecular probes were used at indicated concentrations for Western blot (W), immunofluorescence (IF), or FC: kindlin-2 (MAB2617 from Millipore) W: 1:1,000; talin (8D4 from Sigma-Aldrich) W: 1:1,000; paxillin (610051, clone 349 from BD Transduction Laboratories) IF: 1:500; paxillin (sc-5574 from Santa Cruz Biotechnology) IF: 1:200; integrin β1-Alexa 488-conjugated (102211 from BioLegend) FC: 1:300; integrin β1 (home-made [Azimifar et al, 2012]) W: 1:10,000, IF: 1:4,000, IP: 2 μl/mg lysate; integrin β1 (clone MB1.2 from Millipore) IF: 1:500; integrin β1 (clone JB1B from Novus Biologicals) IP: 5 μl/mg lysate; integrin α5-PE (557446 from Pharmingen) FC: 1:300; IgG2a rat isotype control (13-4321 from eBioscience) FC: 1:300; pT788/789 β1-integrin (44-872G from Thermo Fisher Scientific) W: 1:1,000, IF 1:300; pT788/789 β1-integrin (ab5189 from Abcam) W: 1:1,000, IF 1:300, phospho-threonine (clone 20H6.1 from Merck) W: 1:1,000, IF 1:500, IP: 5 μl/mg lysate; phospho-threonine (clone RM102 from Thermo Fisher Scientific) W: 1:1,000, IF 1:300, IP: 5 μl/mg lysate; PPM1F (Grimm et al, 2020) W: 1:300; SNX17 (10275-1-AP from Proteintech) W: 1:1,500.

The following secondary antibodies were used: goat anti–rabbit Alexa 488-conjugated (A11008), goat anti–mouse Alexa 488-conjugated (A11029), goat anti-rat Alexa 488-conjugated (A11006), goat anti-mouse Alexa 546-conjugated (A11003) (all from Invitrogen), FACS: 1:500, IF: 1:500; goat anti-rat HRP-conjugated (712035150) (both from Dianova) W: 1:10,000, donkey anti-rabbit Cy3-conjugated (711-165-152) (from Jakson ImmunoResearch) IF: 1:500, goat anti-mouse HRP-conjugated (172-1011) and goat anti-rabbit HRP-conjugated (172-1019) (both from Bio-Rad) W: 1:10,000.

Phalloidin-Alexa 647-conjugated (A22287 from Thermo Fisher Scientific) and DAPI (Sigma-Aldrich) were used to stain F-actin and nuclei, respectively.

### Synthetic integrin cytosolic peptides

Synthetic integrin cytosolic peptides were synthesized in house by the Max Planck Institute of Biochemistry Core Facility.

β1-WT peptide (HDRREFAKFEKEKMNAKWDTGENPIYKSAVTTVVNPKYEGK-OH), β1-scrambled peptide (EYEFEPDKVDTGAKGTKMAKNEKKFRNYTVHNIWESRKVAP-OH), β1-TT/AA peptide (HDRREFAKFEKEKMNAKWDTGENPIYKSAVAAVVNPKYEGK-OH), β1-TT/DD peptide (HDRREFAKFEKEKMNAKWDTGENPIYKSAVDDVVNPKYEGK-OH), β1-pTpT peptide (HDRREFAKFEKEKMNAKWDTGENPIYKSAVpTpTVVNPKYEGK-OH), β1-Y783A peptide (HDRREFAKFEKEKMNAKWDTGENPIAKSAVTTVVNPKYEGK-OH), β3/β5-pTpT peptide (KWDTGENPIYKEAVpTpTFTVDFTFNKFNPKYEGK-OH), and Wwc2-pTpT peptide (LLQEKGGYIPSGPIpTpTIHENEVVKSPSQPG-OH).

### Plasmid construction

Point mutations in the mouse β1 integrin tail (TT788/789DD and TT788/789EE) cDNA were introduced by site-directed mutagenesis using the following primers: β1-TTDD fwd CCT ATT TAC AAG AGT GCC GTG gat gaT GTG GTC AAT CCG AAG TAT GAG G; β1-TTDD rev C CTC ATA CTT CGG ATT GAC CAC Atc atc CAC GGC ACT CTT GTA AAT AGG A; β1-TTEE fwd A AAT CCT ATT TAC AAG AGT GCC GTG gaA gaa GTG GTC AAT CCG AAG TAT GAG G; β1-TTEE rev C CTC ATA CTT CGG ATT GAC CAC ttc Ttc CAC GGC ACT CTT GTA AAT AGG ATT. For stably expressing the *β1 integrin* cDNAs, the cDNAs were cloned into the retroviral expression vector *pLZRS*. Retroviral expression constructs of β1-WT, β1-TT788/789AA, β1-Y783A, and β1-Y795A have been described (Böttcher et al, 2012). The correct sequence of all constructs was confirmed by DNA sequencing (Eurofins Genomics).

### Cell lines

Fibroblasts lacking β1 integrin (β1-KO) and reconstituted with β1-WT or β1-TT788/789AA, β1-Y783A, and β1-Y795A have been described (Böttcher et al, 2012). To generate β1-TT/DD and β1-TT/EE reconstituted cell lines, the β1-KO fibroblasts were retrovirally transduced with cDNAs coding for mouse β1-TT/DD or β1-TT/EE and sorted with the flow cytometer to remove non-infected cells. UB epithelial cells were established from the metanephric kidney anlagen of E11.5 mice carrying floxed *β1 integrin* (*Itgb1*^*flox/flox*^) alleles. The T-bud stage UBs were dissected from the metanephric kidney analgen and cultured in 3D-Matrigel in UB culture medium (DMEM/F12 supplemented with 10% charcoal/dextran treated FCS [Hyclone], 200 nM all-trans- and 9-cis retinoic acid [Sigma-Aldrich] and 15–20 ng/ml recombinant rat glial cell-derived neurotrophic factor [R&D Systems]). UB cells were immortalized with SV40 Large-T antigen in the 3D matrix before epithelial UB organoids were dissected and cultured in cell culture dishes in DMEM containing 10% FBS, 5 ng/ml glial cell-derived neurotrophic factor. The UB cells were cloned and the floxed alleles were deleted by adenoviral expression of *Cre* recombinase resulting in β1 integrin knockout cells (β1-KO), and reconstituted with either β1-WT, β1-TT/AA, β1-TT/DD, β1-TT/EE, β1-Y783A, or β1-Y795A. A172 sg-control and A172 PPM1F KO cells have been described (Grimm et al, 2020). All cell lines were cultured in DMEM supplemented with 10% FCS and Penicillin/Streptomycin.

### Transient and stable transfection/transduction

To generate stable cell lines, VSV-G pseudotyped retroviral vectors were produced by transient transfection of 293T (human embryonic kidney) cells. Viral particles were concentrated from cell culture supernatant (Pfeifer et al, 2000) and used for infections. Cells were transiently transfected with Lipofectamine 2000 (Invitrogen) according to the manufacturer’s protocol.

### FC

FC was carried out with a LSRFortessa Flow Cytometer (BD Biosciences) equipped with FACS DiVa software (BD Biosciences). Cells were detached with trypsin/EDTA, sedimented by centrifugation (290*g* for 5 min) and suspended in PBS (P4417; Sigma-Aldrich) containing 1% BSA. 2–4 × 10^5^ cells were incubated with primary antibodies for 1 h on ice, washed with cold PBS supplemented with 1% BSA before incubation with the secondary antibody for 45 min on ice in the dark. Data analysis was carried out with FlowJo (version 9.4.10; BD Biosciences).

### Integrin peptide ELISA

Synthetic integrin cytosolic peptides (β1-WT, β1-TT/AA, β1-TT/DD, β1-pTpT, β3/β5-pTpT, and Wwc2-pTpT) were dissolved in PBS to a concentration of 500 nM and 100 μl was immobilized on 96-well MaxiSorp plates (Nalge Nunc) overnight at 4°C. The plates were blocked with 200 μl PBS containing 3% BSA for 1 h at room temperature and then washed thrice with 200 μl PBS. Different dilutions of the anti–β1-pTpT antibodies (Thermo Fisher Scientific and Abcam) in 100 μl PBS supplemented with 3% BSA were incubated for 2 h at room temperature. After washing thrice with 200 μl PBS, 100 μl of anti-rabbit-IgG HRP-conjugate was added (1:10,000 in PBS supplemented with 3% BS) and incubated for 1 h at room temperature. The plates were washed thrice with 200 μl PBS, developed with 100 μl of ABTS substrate solution (1-Step ABTS; Thermo Fisher Scientific) before measuring absorbance at λ = 405 nm in a SpectraMax ABS Plus plate reader (Molecular Devices).

### Peptide pull-down assays

Pull-down assays were performed with N-terminally desthio biotinylated β1-WT, β1-Scr, β1-TT/AA, β1-TT/DD, β1-pTpT peptides. Before use, peptides were immobilized on 25 μl Dynabeads MyOne Streptavidin C1 (10 mg/ml; Invitrogen) in Mammalian Protein Extraction Reagent (Thermo Fisher Scientific) containing protease inhibitors (cOmplete; Roche) and phosphatase inhibitors (Phosphatase Inhibitor Cocktail 2 and 3; Sigma-Aldrich) for 3 h at 4°C. Cell lysates were prepared with MPER containing protease and phosphatase inhibitors and 0.5 mg of supernatant was incubated with the peptide-coated beads at 4°C under constant rotation. For pull-downs with recombinant proteins, synthetic β1 integrin tail peptides were immobilized on 25 μl Dynabeads MyOne Streptavidin C1 (10 mg/ml; Invitrogen) for 3 h at 4°C, incubated with 2% BSA in Mammalian Protein Extraction Reagent (Thermo Scientific Scientific) for 30 min at 4°C to block unspecific binding before adding kindlin-2 or THD (conc 2.5 and 8.5 nM, respectively) and further incubation on a rotator for 2 h at 4°C. After washing the beads three times with lysis buffer, the proteins bound to the beads were eluted using Laemmli buffer, separated on a 10% SDS–PAGE, and analyzed by Western blotting.

### MST

All MST measurements were performed on a Monolith NT.115 red-blue (Nanotemper) using premium-coated capillaries to reduce non-specific interaction of the proteins with the glass surface. Both interaction partners (ligand and receptor) were transferred into MST buffer (20 mM Tris, pH 7.5, 200 mM sodium chloride, 1 mM tris(2-carboxyethyl)phosphine [TCEP], 0.05% Tween-20) to avoid artifacts derived from buffer mismatches. 200 nM Atto488-labeled integrin β1-WT tails synthesized in house by the Max Planck Institute of Biochemistry Core Facility were used as ligands. The measurements were carried out at 20% light emitting diode power and 20% and 40% MST power. Data were analyzed using the MO Affinity Analysis Software (Nanotemper).

### β1 integrin immunoprecipitations

For immunoprecipitation (IP) of β1 integrin, cells were lysed in β1 lysis buffer (50 mM Tris–HCl, pH 8.0, 150 mM NaCl, 1% Triton X-100, 0.05% sodium deoxycholate [SDC], 10 mM EDTA-containing protease inhibitors [cOmplete; Roche], phosphatase inhibitors [Phosphatase Inhibitor Cocktail 2 and 3; Sigma-Aldrich], and 10 mM phosphatase saturating substrate [para-nitrophenolphosphate [pNPP]; Sigma-Aldrich]) and incubated with rabbit anti-β1 integrin ectodomain antibodies (2 μl/mg cell lysate home-made to IP total [active and inactive] mouse β1 integrin; 5 μg/mg cell lysate JB1B to IP human β1 integrin) for 2 h at 4°C while rotating. Subsequently, lysates were incubated with 50 μl washed protein A/G Plus Agarose (Santa Cruz Biotechnology) for 2 h at 4°C. After repeated washing steps with lysis buffer, proteins were eluted from the beads using Laemmli buffer, separated on a 8% SDS–PAGE and analyzed by Western blotting.

### Atomic force microscopy-based single-cell force spectroscopy

For cell attachment, tip-less V-shaped silicon nitride tip-less cantilevers (NP-O; Bruker) were plasma cleaned (PDC-32G; Harrick Plasma) and then incubated with 2 mg/ml concanavalin A (Sigma-Aldrich) in PBS at 4°C overnight. For substrate coatings, a 200-μm-thick four-segmented polydimethylsilane (PDMS) mask fused to the surface of glass bottom Petri dishes (World Precision Instruments) was used (Yu et al, 2015). Each of the four PDMS framed glass surfaces were incubated with 50 μg/ml FNIII7-10^RGD^ or FNIII7-10^ΔRGD^ in PBS at 4°C overnight. For SCFS, a Nanowizard II AFM equipped with a CellHesion module (both JPK Instruments) was mounted onto an inverted microscope (Observer Z1; Zeiss). The temperature of the medium in the Petri dish was maintained at 37°C (PetriDish heater; JPK Instruments). 200-μm-long cantilevers having nominal spring constants of 0.06 N/m were used. Each cantilever was calibrated prior the measurement by determining its sensitivity and spring constant using the thermal noise analysis using inbuild routines. To adhere a single fibroblast to the AFM cantilever, overnight serum-starved fibroblasts with confluency up to ∼80% were washed with PBS, trypsin/EDTA-detached for 2 min, suspended in SCFS media (DMEM supplemented with 20 mM Hepes) containing 1% (vol/vol) FCS, pelleted, and resuspended in serum free SCFS media. Fibroblasts were allowed to recover for at least 30 min from trypsin treatment (Schubert et al, 2014). Fibroblasts in suspension were incubated with 1 μM cilengitide (Sigma-Aldrich) for 30 min before and throughout the experiments. To attach a single fibroblast to the apex of the cantilever, the fibroblast suspension was pipetted onto the functionalized Petri dishes, the concanavalin A–coated cantilever was lowered onto a single fibroblast at 10 μm/s until a force of 5 nN was recorded and was subsequently retracted after 5 s contact at 10 μm/s for 100 μm. The cantilever-bound fibroblast was incubated for 10–15 min for binding probability experiments and ∼5 min for adhesion force measurements. Using differential interference contract (DIC) microscopy, the morphological state of the fibroblast was monitored. For binding probability experiments, the cantilever-bound fibroblast was lowered onto the substrate at 1 μm/s until a contact force of 200 pN was reached and the cantilever was retracted after 250 ms at constant hight at 1 μm/s for ≥13 μm until the fibroblast and substrate were fully separated. This resulted in a total contact time between fibroblasts and substrate of ∼300 ms. This experimental cycle was repeated for 100 times for each fibroblast, whereby the fibroblast was allowed to recover for 0.5 s between cycles and the contact area on the substrate was altered. FD curves were analyzed for unbinding events using in-build JPK software to determine binding probability as the ratio of FD curves showing unbinding events and total number of attempts. For adhesion force experiments, the rounded cantilever-bound fibroblast was lowered onto the substrate at 5 μm/s until a contact force of 1 nN was recorded. For contact times of 5, 20, 50, or 120 s, the cantilever was maintained at constant height and subsequently retracted at 5 μm/s for 100 μm until the fibroblast and substrate were fully separated. After the experimental cycle, the fibroblast was allowed to recover for a time period equal to contact time before measuring the adhesion force for a different contact time. A single fibroblast was used to probe the adhesion force for all contact times or until morphological changes, that is, spreading, was observed. The sequence of contact time measurements and area of the substrate were varied. Adhesion forces were determined after baseline correction of FD curves with JPK software (JPK Instruments).

### Integrin activation assay

Cells were trypsinized, split into two fractions, and placed on ice. The cell fractions were stained for the 9EG7 epitope, reporting activated β1 integrin or total β1 integrin for 1 h on ice in PBS supplemented with 1% BSA. Cells were washed thrice with PBS and incubated with Alexa 488-conjugated secondary antibody in PBS supplemented with 1% BSA for 45 min on ice in the dark. Cells were washed and fluorescence intensity was measured by FC (BD LSRII, FACSDivaTM software; BD Biosciences).

For ELISA, starved cells were trypsinized and kept in suspension in DMEM supplemented with 3% BSA for 45 min at 37°C before seeding 40,000 cells into FN-coated (10 μg/ml) flat-bottom 96-well plates. After adhering for 40 min at 37°C, the cells were fixed with 2% PFA and washed with PBS. Afterwards, cells were blocked with PBS containing 3% BSA for 60 min at room temperature and stained for the 9EG7 epitope of β1 integrin or total integrin β1 (Millipore) overnight in PBS containing 3% BSA at 4°C. After three washes with PBS, the cells were incubated with the secondary HRP-conjugated goat anti-rat IgG antibody in PBS containing 3% BSA for 1 h at RT. Finally, cells were intensively washed, 100 μl of ABTS substrate solution (1-Step ABTS; Thermo Fisher Scientific) was added and the absorption was measured at λ = 405 nm in a SpectraMax ABS Plus plate reader (Molecular Devices).

### Spreading and adhesion assays

Cells were grown to 70% confluence followed by overnight incubation in DMEM containing 0.2% FCS. After detaching with trypsin/EDTA, cells were serum starved for 1 h at 37°C in adhesion assay medium (10 mM Hepes, pH 7.4; 137 mM NaCl; 1 mM MgCl_2_; 1 mM CaCl_2_; 2.7 mM KCl; 4.5 g/liter Glucose; and 3% BSA [wt/vol]) or DMEM containing 3% BSA in the presence or absence of 5 μM cilengitide.

For adhesion assays, flat-bottom 96-well plates were coated with 10 μg/ml FN (Calbiochem) or 1:500 dilution of Matrigel (Corning) and blocked with PBS containing 3% BSA. Cells were then plated (40,000 per well) in adhesion buffer or DMEM containing 3% BSA ± 5 μM cilengitide and incubated for 15 or 40 min at 37°C. After vigorous washing with PBS to remove nonadherent cells, cells were fixed with PBS containing 4% PFA and adherent cells measured by staining with crystal violet (0.1% in 20% methanol) for 15 min. After extensive washing with H_2_O, the color was released by 2% SDS/H_2_O and the absorption was measured at λ = 570 nm in a SpectraMax ABS Plus plate reader (Molecular Devices).

For cell spreading, 40,000 cells were seeded on 10 μg/ml FN-coated coverslips, cultured in DMEM containing 3% BSA ± 5 μM cilengitide at 37°C, fixed with 4% PFA (wt/vol) in PBS, and stained with phalloidin–Alexa 488 and DAPI. At least 10 images were taken using a Zeiss LSM 780 confocal laser scanning microscope equipped with 40×/NA 1.4 oil objective and cell spreading area was measured using ImageJ (release 1.53 [Schneider et al, 2012]).

### Mass spectrometry analysis

To identify proteins immunoprecipitated with anti-β1-pTpT antibodies, cells were lysed in β1 lysis buffer (50 mM Tris–HCl, pH 8.0, 150 mM NaCl, 1% Triton X-100, 0.05% SDC, 10 mM EDTA containing protease inhibitors [cOmplete; Roche], and phosphatase inhibitors [Phosphatase Inhibitor Cocktail 2 and 3; Sigma-Aldrich]) and incubated with rabbit anti-β1-pTpT antibodies (Thermo Fisher Scientific) for overnight at 4°C while rotating. Subsequently, lysates were incubated with 50 μl washed protein A/G Plus Agarose (Santa Cruz Biotechnology) for 2 h at 4°C. For reduction and alkylation of the proteins, previously PBS-washed beads were incubated with SDC buffer containing 1% SDC (Sigma-Aldrich), 40 mM 2-chloroacetamide (Sigma-Aldrich), 10 mM TCEP (Thermo Fisher Scientific), and 100 mM Tris, pH 8.0, at 37°C. After incubation for 20 min at 37°C, the samples were diluted 1:2 with MS-grade water (VWR). Proteins were digested overnight at 37°C with 0.5 μg trypsin (Promega). Then, the beads material was centrifuged and the supernatant was collected. In addition, the beads were washed with 100 μl of buffer A (0.1% formic acid), followed by centrifugation and collection of the supernatant. The combined supernatants were acidified with TFA (Merck) to a final concentration of 1%. Precipitated SDC was removed by centrifugation and the peptide mixture was desalted via SCX StageTips (Rappsilber et al, 2007). Samples were vacuum-dried and re-suspended in 10 μl of buffer A.

To identify phosphorylated β1 integrin, β1 integrins were immunoprecipitated from β1-WT fibroblasts kept in suspension, seeded for 45 and 90 min on FN or arrested in mitosis as indicated before. Proteins were eluted from the beads using Laemmli buffer, separated on a 8% SDS–PAGE proteins and stained with Coomassie. Gel lanes of interest were excised, chopped, and washed two times with 150 μl of destaining buffer (25 mM ammonium bicarbonate, 50% ethanol). Gel pieces were dehydrated two times in 150 μl of 100% ethanol and the gel pieces were dried by vacuum centrifugation. Then, 50 μl of digestion buffer (25 mM Tris–HCl, 10% acetonitrile, and 10 ng/μl of trypsin) was added. After incubation for 20 min on ice, 50 μl of ammonium bicarbonate buffer (25 mM) was added and the gel pieces were incubated at 37°C overnight. Peptides in the supernatant were collected and more peptides were extracted from the gel pieces by repeated incubation of the gel pieces at 25°C in 100 μl of extraction buffer (3% TFA and 30% acteonitrile), subsequent centrifugation and collection of the supernatants. Finally, the gel pieces were dehydrated by incubation at 25°C in 100 μl of 100% acetonitrile and the supernatant was unified with the supernatants from previous extraction steps. Acetonitrile was removed by vacuum-centrifugation and 70 μl of 2 M Tris–HCl as well as 10 mM TCEP and 40 mM 2-chloroacetamide was added. After incubation for 30 min at 37°C, peptides were acidified to 1% TFA and desalted using StageTips.

### LC MS/MS data acquisition

For anti-β1-pTpT immunoprecipitations: Purified and desalted peptides were loaded onto a 30-cm column (inner diameter: 75 microns; packed in-house with ReproSil-Pur C18-AQ 1.9-micron beads; Dr. Maisch GmbH) via the autosampler of the Thermo Easy-nLC 1000 (Thermo Fisher Scientific). Peptides were loaded in buffer A (0.1% formic acid) at 400 nl/min and percentage of buffer B (80% acetonitrile, 0.1% formic acid) was ramped from 8% to 30% over 35 min followed by a ramp to 35% over 10 min and then 58% over the next 5 min, 95% over the next 5 min, and maintained at 95% for another 5 min.

Data acquisition on the timsTOF Pro (Brucker) was performed using timsControl. The mass spectrometer was operated in data-dependent PASEF mode with one survey thermal ionization mass spectrometry (TIMS)-MS and 10 PASEF MS/MS scans per acquisition cycle. Analysis was performed in a mass scan range from 100 to 1700 m/z and an ion mobility range from 1/K0 = 1.35 to 0.85 Vs cm^−2^ using equal ion accumulation and ramp time in the dual TIMS analyzer of 100 ms each at a spectra rate of 9.43 Hz. Suitable precursor ions for MS/MS analysis were isolated in a window of 2 Th for m/z < 700 and 3 Th for m/z > 700 by rapidly switching the quadrupole position in sync with the elution of precursors from the TIMS device. The collision energy was lowered as a function of ion mobility, starting from 45 eV for 1/K0 = 1.3 Vs cm^−2^ to 27 eV for 0.85 Vs cm^−2^. Singly charged precursor ions were excluded with a polygon filter mask and further m/z and ion mobility information was used for “dynamic exclusion” to avoid re-sequencing of precursors that reached a “target value” of 14,500 a.u. The ion mobility dimension was calibrated linearly using three ions from the Agilent ESI LC/MS tuning mix (m/z, 1/K0: 622.0289, 0.9848 Vs cm^−2^; 922.0097 Vs cm^−2^, 1.1895 Vs cm^−2^; 1221.9906 Vs cm^−2^, and 1.3820 Vs cm^−2^).

To detect phosphorylated β1 integrin: Purified and desalted peptides were loaded onto a 30-cm column (inner diameter: 75 microns; packed in-house with ReproSil-Pur C18-AQ 1.9-micron beads; Dr. Maisch GmbH) via the autosampler of the Thermo Easy-nLC 1000 (Thermo Fisher Scientific) at 60°C. Using the nanoelectrospray interface, eluting peptides were directly sprayed onto the benchtop Orbitrap mass spectrometer Q Exactive HF (Thermo Fisher Scientific).

Peptides were loaded in buffer A (0.1% formic acid) at 250 nl/min and percentage of buffer B (80% acetonitrile, 0.1% formic acid) increased to 30% over 45 min followed by an increase to 60% over 5 min then 95% over the next 5 min. Percentage of buffer B was maintained at 95% for another 5 min. The mass spectrometer was operated in a data-dependent mode with survey scans from 300 to 1,650 m/z (resolution of 60,000 at m/z = 200), and up to 10 of the top precursors were selected and fragmented using higher energy collisional dissociation (HCD with a normalized collision energy of value of 28). The MS2 spectra were recorded at a resolution of 30,000 (at m/z = 200). Automatic gain control target for MS and MS2 scans were set to 3 × 10^6^ and 1 × 10^5^, respectively, within a maximum injection time of 100 and 60 ms for MS and MS2 scans, respectively. Dynamic exclusion was set to 30 ms.

### LC MS/MS data analysis

Raw data were processed using the MaxQuant computational platform (version 1.6.17.0) (Cox & Mann, 2008) with standard settings applied for ion mobility data (Prianichnikov et al, 2020). Shortly, the peak list was searched against the Uniprot database of *Mus musculus* (55,466 entries) with an allowed precursor mass deviation of 10 ppm and an allowed fragment mass deviation of 20 ppm. To detect integrin phosphopeptides, we allowed precursor mass deviation of 4.5 ppm and an allowed fragment mass deviation of 20 ppm.

MaxQuant by default enables individual peptide mass tolerances, which was used in the search. Cysteine carbamidomethylation was set as static modification, and methionine oxidation, phosphorylation (on S, T, and Y), and N-terminal acetylation as variable modifications. The match between the run feature was enabled, and proteins were quantified across samples using the label-free quantification algorithm in MaxQuant as label-free quantification intensities.

### Statistical analysis

All experiments were repeated at least three times (as indicated in figure legends). Most statistical significances (*P < 0.05; **P < 0.01; ***P < 0.001; ****P < 0.0001, ns, not significant) were determined by one-way ANOVA. Two-tailed Mann–Whitney *U* tests were applied to determine significant differences between adhesion forces or the binding probability of fibroblast lines at different conditions, *P*-values comparing adhesion strengthening differences were calculated by two-tailed extra sum of squares F-test and *P*-values comparing rupture force difference were calculated using Kruskal–Wallis test. The statistical analysis was performed using Prism (version 9.00; GraphPad Software) in-build routines.

## Supplementary Material

Reviewer comments
